# Comparison of Multiple State-of-the-Art Large Language Models for Patient Education Prior to CT and MRI Examinations

**DOI:** 10.3390/jpm15060235

**Published:** 2025-06-05

**Authors:** Semil Eminovic, Bogdan Levita, Andrea Dell’Orco, Jonas Alexander Leppig, Jawed Nawabi, Tobias Penzkofer

**Affiliations:** 1 Department of Radiology, Charité–Universitätsmedizin Berlin, Corporate Member of Freie Universität Berlin and Humboldt-Universität zu Berlin, 13353 Berlin, Germany; bogdan.levita@charite.de (B.L.); tobias.penzkofer@charite.de (T.P.); 2 Department of Neuroradiology, Charité–Universitätsmedizin Berlin, Corporate Member of Freie Universität Berlin and Humboldt-Universität zu Berlin, 13353 Berlin, Germany; andrea.dellorco@charite.de (A.D.); jawed.nawabi@charite.de (J.N.); 3 Department of Radiology, Immanuel Klinik Rüdersdorf—Universitätsklinikum der Medizinischen Hochschule Brandenburg, 15562 Rüdersdorf bei Berlin, Germany; jonas.leppig@immanuelalbertinen.de; 4 Berlin Institute of Health, 10178 Berlin, Germany

**Keywords:** artificial intelligence, large language model, computed tomography, magnetic resonance imaging

## Abstract

**Background/Objectives**: This study compares the accuracy of responses from state-of-the-art large language models (LLMs) to patient questions before CT and MRI imaging. We aim to demonstrate the potential of LLMs in improving workflow efficiency, while also highlighting risks such as misinformation. **Methods**: There were 57 CT-related and 64 MRI-related patient questions displayed to ChatGPT-4o, Claude 3.5 Sonnet, Google Gemini, and Mistral Large 2. Each answer was evaluated by two board-certified radiologists and scored for accuracy/correctness/likelihood to mislead using a 5-point Likert scale. Statistics compared LLM performance across question categories. **Results**: ChatGPT-4o achieved the highest average scores for CT-related questions and tied with Claude 3.5 Sonnet for MRI-related questions, with higher scores across all models for MRI (ChatGPT-4o: CT [4.52 (± 0.46)], MRI: [4.79 (± 0.37)]; Google Gemini: CT [4.44 (± 0.58)]; MRI [4.68 (± 0.58)]; Claude 3.5 Sonnet: CT [4.40 (± 0.59)]; MRI [4.79 (± 0.37)]; Mistral Large 2: CT [4.25 (± 0.54)]; MRI [4.74 (± 0.47)]). At least one response per LLM was rated as inaccurate, with Google Gemini answering most often potentially misleading (in 5.26% for CT and 2.34% for MRI). Mistral Large 2 was outperformed by ChatGPT-4o for all CT-related questions (*p* < 0.001) and by ChatGPT-4o (*p* = 0.003), Google Gemini (*p* = 0.022), and Claude 3.5 Sonnet (*p* = 0.004) for all CT Contrast media information questions. **Conclusions**: Even though all LLMs performed well overall and showed great potential for patient education, each model occasionally displayed potentially misleading information, highlighting the clinical application risk.

## 1. Introduction

Computed tomography (CT) and magnetic resonance imaging (MRI) are critical diagnostic tools extensively used in medical facilities worldwide. Overall, the demand for CT and MRI examinations continues to increase in line with population growth and demographic change [1].

However, the use of both modalities varies greatly worldwide due to socioeconomic and political factors. From clinical experience, patients’ knowledge of CT and MRI examinations is often inadequate [2,3]. There is usually a great need for knowledge regarding the use of contrast media and the risks of the examination. In addition, examinations of children and pregnant patients often lead to uncertainty and insecurity. Despite their routine nature, these imaging procedures require comprehensive patient education and informed consent, which often consumes significant time and resources within the hospital setting. While the focus extends beyond efficiency, it increasingly prioritizes supporting patient empowerment, potentially contributing to better compliance by reducing uncertainty and fear.

As medical facilities strive to optimize efficiency with the goal of optimal patient care, there is growing interest in leveraging advanced technologies to streamline different processes such as more efficient patient education. One promising approach is the utilization of large language models (LLMs), which are capable of providing detailed, accurate responses to patient inquiries [4,5,6].

It is not yet certain how safely LLMs can be utilized in obtaining patient-relevant information prior to a CT or MRI examination and to what extent LLM-generated responses can effectively reduce the burden of patient education in clinical settings, improve patient satisfaction, and streamline workflow. With many models improving rapidly and greatly over short periods of time, it is necessary to compare those in terms of accuracy when addressing radiology-related inquiries.

This study seeks to evaluate the potential of LLMs in enhancing patient education by comparing their accuracy in answering frequently asked questions related to CT and MRI imaging. By systematically assessing the accuracy of responses generated by four different state-of-the-art models, we aim to compare LLMs for clinical use. The findings could significantly impact how patient interactions are managed in radiology departments, potentially improving workflow efficiency and patient satisfaction.

## 2. Materials and Methods

### 2.1. General Study Design

Due to the human-generated sample dataset, it was not mandatory to obtain a positive ethics vote from Charité’s Ethics Committee. In this prospective evaluation study with a controlled experimental design, two radiology residents (second-year resident, S.E.; fifth-year resident, B.L.) created a set of questions primarily reflecting commonly asked patient questions encountered in daily clinical practice before undergoing CT and MRI imaging, and questions adapted from the official consent forms [7,8] that patients receive prior to the examination (121 questions: 57 questions for CT and 64 for MRI). Responses to these questions provided by four state-of-the-art, closed-source LLMs (ChatGPT 4o, OpenAI; Google Gemini, Google DeepMind; Claude 3.5 Sonnet, Anthropic; Mistral Large 2, Mistral AI) were evaluated independently by two board-certified radiologists with six- and seven-years clinical experience (J.A.L. and J.N., respectively), using a 5-point Likert scale (Supplementary Table S1). These four models were selected in particular as they are well known in the general public, scientifically validated, and overall among the most powerful models.

### 2.2. Question Design and Prompting

Question categories for CT-related questions were general and technical information, contrast media information (general information and side effects, allergic reaction, extravasation, kidney, thyroid gland), pregnancy, breastfeeding and pediatric information, pre- and post-procedure information; for MRI-related questions general and technical information, information about external material, contrast media information, pregnancy, breastfeeding and pediatric information, pre- and post-procedure information. The following is an example of a patient question that was asked concerning the kidney function as indicated by the glomerular filtration rate (GFR):

*“My kidney function is impaired with a GFR of about 45—can I have a CT with contrast?” or “I have a pacemaker—can I still have an MRI?”*

We zero-shot prompted all four LLMs in the English language in the exact same way with

*“I am a patient. I am due to have a CT scan and have some questions about this examination. Can you answer each of the following questions in an understandable way.” and, ”I am a patient. I am due to have an MRI scan and have some questions about this examination. Can you answer each of the following questions in an understandable way.”*

We used a zero-shot prompting approach to reflect realistic usage scenarios, where patients typically seek information without prior domain knowledge or detailed instruction. This approach aligns with the goal of assessing LLMs’ capability to respond intuitively to unstructured, naturally phrased questions that may arise in preparation for CT or MRI examinations.

Afterward, all questions for CT and for MRI were each submitted in the exact same way as a list in a single request in one chat session via public web interface to all four LLMs (on 4 September 2024) to ensure comparability and consistency. This batch prompting approach ensured consistency and comparability across models. The standardized input method allowed for a systematic evaluation of LLM performance across a varied set of questions. All questions are provided as Supplementary Materials, and all responses are also documented.

### 2.3. Response Evaluation

**Figure 1:**
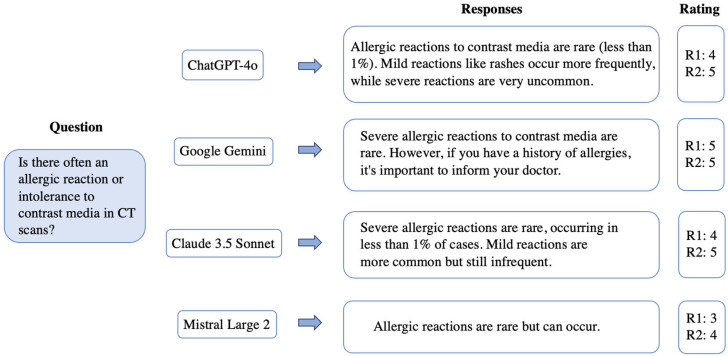
Display of an exemplary question on contrast-agent side effects with the specific responses of each LLM based on our prompt. Legend: R1, radiologist 1; R2, radiologist 2.

Accuracy/correctness of responses provided by ChatGPT 4o, Google Gemini, Claude 3.5 Sonnet, and Mistral Large 2 were rated by two independent and board-certified radiologists using a 5-point Likert scale [9] (1 = Very inaccurate/completely false, very likely to mislead; 2 = Inaccurate/mostly false, likely to mislead; 3 = Neutral/moderately accurate, overall acceptable; 4 = Accurate/mostly correct, only very few inaccuracies, unlikely to mislead; 5 = Very accurate/completely correct, very unlikely to mislead; displayed in Supplementary Table S1) and were then statistically compared. The LLMs were anonymized so that the radiologists did not know which LLM they were evaluating. Figure 1 shows an example of a question on contrast-agent side effects, the responses of each LLM, and the ratings of both radiologists. There were no explicit restrictions placed on the response length.

### 2.4. Statistical Analysis

For the display of categorical ordinal data, mean and standard deviation were used. The overall mean was calculated from the mean values of the two radiologists’ assessments. For testing on statistically significant differences between the models, we applied a two-tailed, four sampled Friedman test based on our repeated measures and the ordinal scale. *p*-values less than 0.05 were considered significant. If so, a post hoc Wilcoxon signed-rank test with a Holm correction was performed to investigate whether the differences between the ratings of each couple of LLMs was statistically significant. The Holm correction was applied to adjust the significance level for multiple comparisons.

Interrater agreement was assessed using the two-way random effects, single-measure intraclass correlation coefficient to evaluate absolute agreement between both raters across all items. Analyses were performed and graphs and diagrams were created using Python (version 3.9.13) with multiple packages including Pandas (version 2.2.2), NumPy (version 1.23.1), SciPy (version 1.13.1; Scikit-Posthocs Version 0.10.0), Matplotlib (version 3.9.2), and Seaborn (version 0.13.2). The applied hypothesis tests were implemented in Python using *scipy.stats.friedmanchisquare* for the Friedman test and *scipy.stats.wilcoxon* for pairwise Wilcoxon signed-rank tests, with Holm correction via *statsmodels.stats.multitest.multipletests*.

## 3. Results

**Figure 2:**
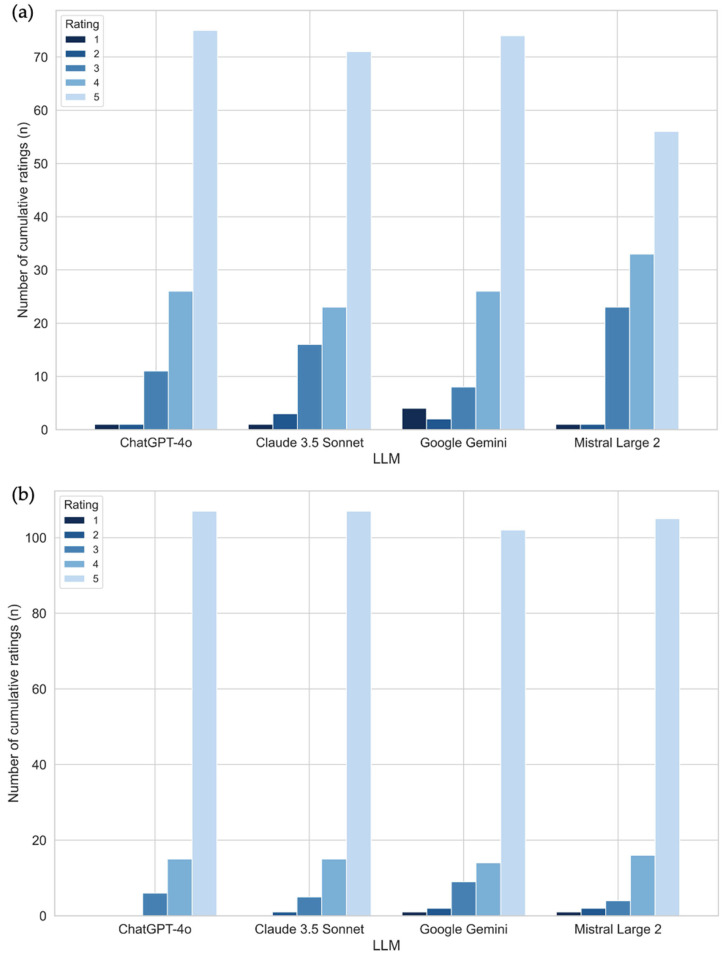
Bar charts that display the cumulative grading of the performance of four LLMs (ChatGPT 4o, Google Gemini, Claude 3.5 Sonnet, Mistral Large 2) assessed by two radiologists in answering patient questions before a CT (**a**) and MRI (**b**) examination.

For CT-related questions, ChatGPT-4o achieved the highest average score of [4.52 (±0.46)], followed closely by Google Gemini [4.44 (±0.58)] and Claude 3.5 Sonnet [4.40 (±0.59)]. Mistral Large 2 scored lower, with an average of [4.25 (±0.54)] (Table 1). Ratings 1 (very likely to mislead) and 2 (likely to mislead) are especially critical regarding misinformation: For CT-related questions four answers from Google Gemini and one answer from each of the other three LLMs were rated as very inaccurate and likely to mislead (Table 1). An exemplary misleading answer was the following: Google Gemini answered the question “I have a pacemaker—can I have a CT scan with contrast medium?” with “Generally, a CT scan with contrast agent is possible in patients with a pacemaker. However, there are certain types of pacemakers that can interact with strong magnetic fields. Consult with your doctor for specific advice.” This is misleading as CT scanners do not generate magnetic fields with Google Gemini mixing up MRI and CT safety issues. Three responses from Claude 3.5 Sonnet, two from Google Gemini, and one from each ChatGPT 4o and Mistral Large 2 were rated with a rating of 2 as inaccurate and still likely to mislead (Table 1; Supplementary Table S2 provides separate CT ratings for each radiologist). The percentage of the lowest rating for CT-related questions (score 1 and 2) was highest for Google Gemini with 5.26% of all responses, 3.51% for Claude 3.5 Sonnet, and 1.76% for both ChatGPT 4o and Mistral Large 2. Figure 2 displays the distribution of the ratings for each LLM.

For MRI-related questions, the average scores were generally higher across all models: ChatGPT-4o [4.79 (±0.37)] and Claude 3.5 Sonnet [4.79 (±0.37)] both achieved equally high average scores, while Mistral Large 2 and Google Gemini followed closely with averages of 4.74 (±0.47) and 4.68 (±0.58), respectively. Furthermore, the proportion of answers that were scored with ratings 1 or 2 was also lower compared to the CT-related questions: Google Gemini and Mistral Large both answered three questions (very) inaccurate (2.40%), Claude 3.5 Sonnet only responded one time (0.78%), and ChatGPT 4o not once (0.0%) in a misleading way (Table 2; Supplementary Table S3 provides separate MRI ratings for each radiologist). An exemplary accurate answer to the question “I am breastfeeding and am to have a contrast agent for an MRI—can this have side effects for me or the baby?” was the following from Google Gemini: “Some contrast agents may pass into breast milk. It’s best to consult with your doctor about the specific contrast agent you will be using and the potential risks for you and your baby. They may recommend pumping and discarding breast milk for a certain period after the scan.” as it explains the possibility of a risk and in particular points out that this should be discussed again with the attending physician. The ratings are shown in Table 1 for the CT-related questions and in Table 2 for the MRI-related questions.

Table 3 demonstrates significant differences between the four LLMs for all CT-related questions (*p* < 0.001) and for following question categories: general and technical information (*p* = 0.009), contrast media information (*p* < 0.001) and contrast media information—thyroid gland (*p* = 0.007).

In contrast, all models performed without statistically significant differences for MRI-related questions (Table 4; Supplementary Table S4 provides a comparative analysis). Mistral Large 2 was outperformed by ChatGPT-4o for all CT-related questions (*p* < 0.001) and by ChatGPT-4o (*p* = 0.003), Google Gemini (*p* = 0.022), and Claude 3.5 Sonnet (*p* = 0.004) for all CT contrast media information questions (*p* < 0.03) (Table 5). Interrater agreement for both radiologists (measured by intraclass correlation coefficient (ICC)) ranged from 0.006 to 0.071 for CT and from –0.026 to 0.260 for MRI across all models (Supplementary Table S5).

## 4. Discussion

In this study, we investigated the performance of four advanced LLMs related to relevant and frequently asked patient questions before CT and MRI examinations. Our primary objective was to evaluate how efficient these LLMs provide important information to patients about their imaging procedures. As a secondary objective we compared different models, especially since there are chargeable and free models.

In general, all four LLMs performed well in answering CT- and MRI-related questions. The models covered key areas very well, such as general or technical information, preparation for an examination, use of contrast media, potential risks and complications, special precautions during pregnancy and breastfeeding, and aftercare. Interestingly the average scores for answering MRI-related questions were generally higher across all models. There were significant differences in how the models handled CT-related questions. This indicates that while all models have strengths, their performance varies in specific medical contexts. ChatGPT-4o consistently performed well across most question categories. In contrast, Mistral Large 2 struggled with CT-related questions, while Google Gemini had weaker performance in MRI-related questions.

However, in patient education, avoiding misleading or harmful information is more important than producing a perfect response. Therefore, we assessed how often each LLM provided potentially misleading answers. Every LLM was rated as inaccurate or very inaccurate for at least one response; only ChatGPT-4o was able to avoid this rating for the MRI-related questions. Google Gemini, for instance, received this rating for six CT-related and three MRI-related answers, e.g., answering the question “Can contrast media in a CT scan have side effects on the thyroid gland?” with “The contrast agents used in CT scans generally do not directly affect the thyroid gland.” Even though Google Gemini answered further and also more specific questions regarding the thyroid gland very well, those individual outliers can potentially misinform patients, thus showing that a consultation with a doctor is currently still essential. When a response was rated inaccurate, it usually came from only one or two of the models for that particular question. One question where all models gave inaccurate responses was: “Do I have to drink a lot after the CT scan with contrast medium?” All models agreed, stating that increased fluid intake helps to eliminate the contrast agent. While not entirely incorrect, this advice lacks clear evidence for patients without a kidney disease or other risk factors [10,11], and may cause unnecessary overhydration. This highlights the importance of patient-specific context, especially for complex or scientifically debated topics, where generic advice may not be appropriate.

LLMs differ substantially from conventional FAQ systems primarily due to their ability to generate personalized responses, address specific questions, and offer detailed, context-aware information. The overall very good results of ChatGPT-4o are in line with the literature [12,13,14,15,16,17] and reflect a highly advanced LLM. ChatGPT’s increased collaboration with medical experts and companies appears to be reaping rewards [15,16]. Besides our study, numerous research projects in various medical specialties have shown the benefits of LLMs, particularly in radiology [17,18] LLMs are used to improve diagnostic accuracy, assist in training of students and doctors, and translate complex findings into clear and understandable language for patients. As noted, LLMs in radiology help patients better understand medical terms and improve consent discussions [13,19,20,21]. Studies have shown the potential of LLMs in answering patient questions in fields like ophthalmology [22], anesthesiology [23], dermatology [24], ENT [25], and plastic surgery [26], supporting our findings in radiology education.

A study by Armbruster et al. [19] found ChatGPT-4.0 responses rated higher in usefulness and empathy in answering patient questions than those of a medical expert panel. However, unlike medical experts, patients were unable to distinguish harmless from potentially harmful advice from ChatGPT-4.0, which poses an important risk. This aspect cannot be emphasized often enough [17], especially in a medical context—as stated above, our findings underline this important point. Regardless of the LLM quality, doctors must review responses to ensure patients receive complete and safe information [19]. Still, it can be assumed that doctors must have in-depth knowledge of a medical specialty in order to recognize risky advice from LLMs [27]. Transparent communication about LLMs is essential to raise patient awareness about their purpose, advantages, and risks.

There are also concerns about ChatGPT’s consistency. Chen et al. [28] demonstrated that performance between ChatGPT-3.5 and ChatGPT-4 varied greatly within just three months, stressing the need for continued monitoring. Future LLM development in healthcare must include safeguards to prevent misinformation and ensure responses follow evidence-based guidelines and peer-reviewed clinical knowledge. Systematic evaluations of open-source models are needed to assess their reliability and reduce the risk of spreading incorrect health information.

The patient questions in this study were generated using zero-shot prompting. More advanced methods like few-shot or in-context learning were not utilized, which may limit the generalizability of the results. However, this approach better reflects real-life use, as patients do not typically use complex prompts. This prompting method also supports accessibility for patients with limited digital or language skills and provides a baseline for LLM performance.

The LLMs analyzed in this study are closed-source models, which can raise critical concerns about transparency, reliability, and data security [29,30]. This poses a significant challenge for clinical transferability, as those models might need to navigate additional regulatory and confidentiality hurdles to ensure compliance with patient data protection standards. With ChatGPT-4o and Claude 3.5 Sonnet being paid services, free models like Google Gemini and Mistral Large 2 may become more popular among patients. Given how quickly LLMs are evolving, free models may soon offer similar quality and reliability.

LLMs also raise ethical concerns, including legal responsibility and data protection [18,31]. The allocation of responsibility in the event of potential patient harm can be very complex [32]. Furthermore, data collection through LLMs may compromise patient privacy [33], disrupt the doctor–patient relationship, and reduce trust in doctors. However, using LLMs for consent processes may enhance patient understanding and support patient decision-making [34]. Patient safety should continue to be prioritized in the future of LLM development. This makes it necessary to create ethical guidelines that ensure the responsible use of LLMs [33]. Technical and privacy challenges must be addressed through ongoing monitoring and validation.

Submitting all CT and MRI questions at once helps the LLM maintain consistent responses. However, this can reduce personalization and detail compared to alternative prompting methods like chain-of-thought prompting, clarification-first prompting, or multi-turn prompting. By presenting all questions at once, the model tends to provide concise, focused answers, which can streamline the reading process and support in quickly identifying the most essential information. However, the responses may become noticeably shorter, as the model tends to compress its output when handling multiple queries at once, potentially omitting detail and depth.

Our findings demonstrate that LLMs currently can still provide misleading responses on medical topics, underscoring the necessity for physician consultation to remain indispensable in ensuring accurate patient education and care. Nonetheless, LLMs could improve patient interactions by reducing anxiety and increasing compliance.

## 5. Limitations

Interrater agreement, as measured by the ICC, was generally low across all models, indicating considerable variability in how radiologists rated the quality of the LLM-generated responses. This variability may be due to subjective interpretation of partially correct responses on a 5-point Likert scale, especially in a medical context where subtle differences in phrasing can influence interpretation. Improved interrater reliability could be achieved by creating more specific rating criteria or conducting calibration sessions.

While our study simulated written patient interactions, the results cannot be directly transferred to a real doctor–patient conversation. Real interactions include non-verbal cues, follow-up questions, and cultural context-factors LLMs struggle to replicate. This limits the generalizability of our findings. Our study involved no real patients, so it remains unclear how patients would formulate questions or interpret the responses. The questions were created by two radiology residents and evaluated by two expert radiologists, but input from patients would also be valuable. The lack of a standardized framework for question development may limit the generalizability of the study findings, as the selection was based primarily on clinical experience and consent forms.

We focused on accuracy, but other important evaluation metrics in patient education include clarity and empathy. Future studies should assess how consistently LLMs answer and the validity of questions. Another potential limitation is the subjectivity of the response ratings, as those are depending on the experts’ personal experience, expertise, expectations, and evaluation standards. Transfer to clinical practice remains limited due to unclear accountability for harmful advice. Despite these limitations, our findings provide a solid base for future research, which would ideally involve actual patients.

## 6. Conclusions

Even though all four LLMs and especially ChatGPT-4o performed well overall in answering CT- and MRI-related questions for patient education, each model occasionally displayed potentially misleading information, which highlights the application risks in clinical patient interaction. This demonstrates that they cannot (yet) replace a detailed consultation with a doctor and should be constantly validated. However, they still have the potential to reshape the future of radiology services and promote improvements in patient care. Further studies should evaluate the consistency of our results and facilitate the establishment of LLMs in clinical practice. Moreover, further research should integrate patient feedback during model development; prioritize the evaluation of patient understanding, including real-world testing of how patients interpret LLM-generated advice; and, especially, test LLM performance in real-time clinical workflows.

## Figures and Tables

**Table 1:** Grading the performance of four large language models (ChatGPT 4o, Google Gemini, Claude 3.5 Sonnet, Mistral Large 2) in answering patient questions before a CT examination (evaluations of both radiologists combined), with two radiologists grading 57 CT-related questions, 114 ratings in total.

Rating	ChatGPT 4o	Google Gemini	Claude 3.5 Sonnet	Mistral Large 2
5	75 (65.79%)	74 (64.91%)	71 (62.28%)	56 (49.12%)
4	26 (22.81%)	26 (22.81%)	23 (20.18%)	33 (28.95%)
3	11 (9.65%)	8 (7.02%)	16 (14.04%)	23 (20.18%)
2	1 (0.88%)	2 (1.75%)	3 (2.63%)	1 (0.88%)
1	1 (0.88%)	4 (3.51%)	1 (0.88%)	1 (0.88%)
Mean (±SD)	4.52 (±0.46)	4.44 (±0.58)	4.40 (±0.59)	4.25 (±0.54)

Legend: SD, standard deviation.

**Table 2:** Grading the performance of four large language models (ChatGPT 4o, Google Gemini, Claude 3.5 Sonnet, Mistral Large 2) in answering patient questions before an MRI examination (evaluations of both radiologists combined), with two radiologists grading 64 MRI-related questions, 128 ratings in total.

Rating	ChatGPT 4o	Google Gemini	Claude 3.5 Sonnet	Mistral Large 2
5	107 (83.59%)	102 (79.69%)	107 (83.59%)	105 (82.03%)
4	15 (11.72%)	14 (10.94%)	15 (11.72%)	16 (12.5%)
3	6 (4.69%)	9 (7.03%)	5 (3.91%)	4 (3.13%)
2	0 (0.0%)	2 (1.56%)	1 (0.78%)	2 (1.56%)
1	0 (0.0%)	1 (0.78%)	0 (0.0%)	1 (0.78%)
Mean (±SD)	4.79 (±0.37)	4.68 (±0.58)	4.79 (±0.37)	4.74 (±0.47)

Legend: SD, standard deviation.

**Table 3:** Comparative analysis of the performance of four large language models (ChatGPT 4o, Google Gemini, Claude 3.5 Sonnet, Mistral Large 2) in answering patient questions before a CT examination.

	ChatGPT 4o	Google Gemini	Claude 3.5 Sonnet	Mistral 2.0	*p*-Value Friedman Test
All questions (mean, SD)	4.52 (±0.46)	4.44 (±0.58)	4.40 (±0.59)	4.25 (±0.54)	<0.001 **
General and technical information (mean, SD)	4.92 (±0.19)	4.96 (±0.14)	4.62 (±0.58)	4.69 (±0.38)	0.009 **
Contrast media information (mean, SD)	4.48 (±0.39)	4.44 (±0.50)	4.46 (±0.40)	4.06 (±0.55)	<0.001 **
General Information and side effects (mean, SD)	4.33 (±0.41)	4.17 (±0.61)	4.17 (±0.41)	4.00 (±0.63)	0.557 (n.s.)
Allergic reaction (mean, SD)	4.75 (±0.29)	4.75 (±0.29)	4.88 (±0.25)	4.25 (±0.50)	0.097 (n.s.)
Extravasation (mean, SD)	5.00 (±0.00)	4.83 (±0.29)	4.83 (±0.29)	4.67 (±0.29)	0.262 (n.s.)
Kidney (mean, SD)	4.42 (±0.38)	4.50 (±0.32)	4.50 (±0.32)	4.25 (±0.52)	0.145 (n.s.)
Thyroid gland (mean, SD)	4.29 (±0.27)	4.29 (±0.57)	4.29 (±0.27)	3.57 (±0.19)	0.007 **
Pregnancy, breastfeeding, and pediatric examinations (mean, SD)	4.50 (±0.35)	4.28 (±0.62)	4.28 (±0.94)	4.28 (±0.44)	0.400 (n.s.)
Pre- and post-examination information (mean, SD)	4.06 (±0.58)	3.83 (±0.56)	4.06 (±0.53)	4.11 (±0.42)	0.969 (n.s.)

Legend: SD, standard deviation. **: *p* < 0.010; n.s.: *p* > 0.050.

**Table 4:** Comparative analysis of the performance of four large language models (ChatGPT 4o, Google Gemini, Claude 3.5 Sonnet, Mistral Large 2) in answering patient questions before an MRI examination.

	ChatGPT 4o	Google Gemini	Claude 3.5 Sonnet	Mistral Large 2	*p*-Value Friedman Test
All questions (mean, SD)	4.79 (±0.37)	4.68 (±0.58)	4.79 (±0.37)	4.74 (±0.47)	0.173 (n.s.)
**Categories**					
General and technical information (mean, SD)	4.90 (±0.31)	4.83 (±0.49)	4.85 (±0.33)	4.90 (±0.21)	0.456 (n.s.)
Information about external material (mean, SD)	4.72 (±0.45)	4.69 (±0.48)	4.81 (±0.25)	4.84 (±0.24)	0.531 (n.s.)
Contrast media information (mean, SD)	4.68 (±0.37)	4.50 (±0.71)	4.68 (±0.46)	4.46 (±0.63)	0.127 (n.s.)
Pregnancy, breastfeeding, and pediatric examinations (mean, SD)	5.00 (±0.00)	4.83 (±0.41)	4.83 (±0.41)	4.75 (±0.42)	0.262 (n.s.)
Pre- and post-examination information (mean, SD)	4.64 (±0.38)	4.36 (±0.80)	4.64 (±0.48)	4.50 (±0.76)	0.491 (n.s.)

Legend: SD, standard deviation; n.s.: *p* > 0.050.

**Table 5:** Display of *p*-values for the Wilcoxon signed-rank test with Holm correction for those question categories for which statistically significant differences in response gradings were assessed.

*p*-Values Wilcoxon Signed-Rank Test with Holm Correction				
	ChatGPT 4o	Google Gemini	Claude 3.5 Sonnet	Mistral Large 2
CT—All questions				
ChatGPT 4o	-	0.903 (n.s.)	0.299 (n.s.)	<0.001 **
Google Gemini	-	-	0.903 (n.s.)	0.071 (n.s.)
Claude 3.5 Sonnet	-	-	-	0.058 (n.s.)
Mistral Large 2	-	-	-	-
CT—General and technical information				
ChatGPT 4o	-	0.635 (n.s.)	0.170 (n.s.)	0.190 (ns)
Google Gemini	-	-	0.118 (n.s.)	0.170 (ns)
Claude 3.5 Sonnet	-	-	-	0.914 (ns)
Mistral Large 2	-	-	-	-
CT—Contrast media information				
ChatGPT 4o	-	1.000 (n.s.)	1.000 (n.s.)	0.003 **
Google Gemini	-	-	1.000 (n.s.)	0.022 *
Claude 3.5 Sonnet	-	-	-	0.004 **
Mistral Large 2	-	-	-	-
CT—Contrast media information—thyroid gland				
ChatGPT 4o	-	1.000 (n.s.)	1.000 (n.s.)	0.097 (n.s.)
Google Gemini	-	-	1.000 (n.s.)	0.328 (n.s.)
Claude 3.5 Sonnet	-	-	-	0.097 (n.s.)
Mistral Large 2	-	-	-	-

*: significant (*p* < 0.050); **: *p* < 0.010; n.s.: *p* > 0.050; n.s.: *p* > 0.050

## Data Availability

The original contributions presented in this study are included in the article/Supplementary Materials. Further inquiries can be directed to the corresponding author.

## Supplementary Materials

The following supporting information can be downloaded at: https://www.mdpi.com/article/10.3390/jpm15060235/s1, Table S1: Likert-scale for radiologist evaluation of LLM response quality (accuracy/correctness/likelihood to mislead); Table S2: Grading of the performance of four Large Language Models (ChatGPT 4o, Google Gemini, Claude Sonnet 3.5, Mistral Large 2) in answering 57 patient questions before a CT examination (evaluations of both radiologists separated); Table S3: Grading of the performance of four Large Language Models (ChatGPT 4o, Google Gemini, Claude Sonnet 3.5, Mistral Large 2) in answering patient questions before an MRI examination (evaluations of both radiologists separated); Table S4: Display of p values of Wilcoxon Signed-Rank test with Holm correction for all MRI categories; Table S5: Display of Interrater Agreement for both radiologists (Intraclass Correlation Coefficient (ICC)) per model for CT and MRI.

## Abbreviations

The following abbreviations are used in this manuscript:

LLM	Large language model
CT	Computed tomography
MRI	Magnetic resonance imaging
GFR	Glomerular filtration rate
IQR	Interquartile Range
R1	Radiologist 1
R2	Radiologist 2
ICC	Intraclass correlation coefficient
