# Supplementary material for: Comparison of Multiple State-of-the-Art Large Language Models for Patient Education Prior to CT and MRI Examinations
Source: J Pers Med. 2025 Jun 5;15(6):235. doi: 10.3390/jpm15060235 (PMC12194482; doi:10.3390/jpm15060235)
Supplement: Supplementary file 1 [file jpm-15-00235-s001.zip › jpm-3630403-supplementary.pdf]

Supplementary Table S1 Likert-scale for radiologist evaluation of LLM response quality (accuracy / correctness / likelihood to mislead)

Rating	Description
5	Very accurate / completely correct, very unlikely to mislead
4	Accurate / mostly correct, only very few inaccuracies, unlikely to mislead
3	Neutral / moderately accurate, overall acceptable
2	Inaccurate / mostly false, likely to mislead
1	Very inaccurate / completely false, very likely to mislead

Supplementary Table S2 Grading of the performance of four Large Language Models (ChatGPT 4o, Google Gemini, Claude Sonnet 3.5, Mistral Large 2) in answering 57 patient questions before a CT examination (evaluations of both radiologists separated).

**Rating**	**ChatGPT 4o**		**Google Gemini**		**Claude 3.5 Sonnet**		**Mistral Large 2**	
	R1	R2	R1	R2	R1	R2	R1	R2
5	21 (36.84%)	54 (94.74%)	20 (35.09%)	54 (94.74%)	22 (38.6%)	49 (85.96%)	14 (24.56%)	42 (73.68%)
4	23 (40.35%)	3 (5.26%)	23 (40.35%)	3 (5.26%)	17 (29.82%)	6 (10.53%)	18 (31.58%)	15 (26.32%)
3	11 (19.3%)	0 (0.0%)	8 (14.04%)	0 (0.0%)	14 (24.56%)	2 (3.51%)	23 (40.35%)	0 (0.0%)
2	1 (1.75%)	0 (0.0%)	2 (3.51%)	0 (0.0%)	3 (5.26%)	0 (0.0%)	1 (1.75%)	0 (0.0%)
1	1 (1.75%)	0 (0.0%)	4 (7.02%)	0 (0.0%)	1 (1.75%)	0 (0.0%)	1 (1.75%)	0 (0.0%)
Average	4.09 / 5	4.95 / 5	3.93 / 5	4.95 / 5	3.98 / 5	4.82 / 5	3.75 / 5	4.74 / 5

Legend: R1, radiologist 1; R2, radiologist 2.

Supplementary Table S3 Grading of the performance of four Large Language Models (ChatGPT 4o, Google Gemini, Claude Sonnet 3.5, Mistral Large 2) in answering patient questions before an MRI examination (evaluations of both radiologists separated).

**Rating**	**ChatGPT 4o**		**Google Gemini**		**Claude 3.5 Sonnet**		**Mistral Large 2**	
	R1	R2	R1	R2	R1	R2	R1	R2
5	47 (73.44%)	60 (93.75%)	44 (68.75%)	58 (90.63%)	46 (71.88%)	61 (95.31%)	46 (71.88%)	59 (92.19%)
4	12 (18.75%)	3 (4.69%)	10 (15.63%)	4 (6.25%)	13 (20.31%)	2 (3.13%)	12 (18.75%)	4 (6.25%)
3	5 (7.81%)	1 (1.56%)	7 (10.94%)	2 (3.13%)	4 (6.25%)	1 (1.56%)	4 (6.25%)	0 (0.0%)
2	0 (0.0%)	0 (0.0%)	2 (3.13%)	0 (0.0%)	1 (1.56%)	0 (0.0%)	1 (1.56%)	1 (1.56%)

1	0 (0.0%)	0 (0.0%)	1 (1.56%)	0 (0.0%)	0 (0.0%)	0 (0.0%)	1 (1.56%)	0 (0.0%)
Average	4.66 / 5	4.92 / 5	4.47 / 5	4.88 / 5	4.63 / 5	4.94 / 5	4.58 / 5	4.89 / 5

Legend: R1, radiologist 1; R2, radiologist 2.

Supplementary Table S4 Display of p values of Wilcoxon Signed-Rank test with Holm correction for all MRI categories

**P values Wilcoxon Signed-Rank test with Holm correction**				
	ChatGPT 4o	*Google Gemini*	*Claude 3.5 Sonnet*	*Mistral Large 2*
**MRI -** All Questions				
ChatGPT 4o	**-**	0.242 (n.s.)	0.757 (n.s.)	0.757 (n.s.)
Google Gemini	-	-	0.074 (n.s.)	0.731 (n.s.)
Claude 3.5 Sonnet	-	-	-	0.731 (n.s.)
Mistral Large 2	-	-	-	-
**MRI -** General and technical information				
ChatGPT 4o	**-**	0.944 (n.s.)	0.944 (n.s.)	1.000 (ns)
Google Gemini	-	-	1.000 (n.s.)	1.000 (ns)
Claude 3.5 Sonnet	-	-	-	0.944 (ns)
Mistral Large 2	-	-	-	-
**MRI –** Information about external Material				
ChatGPT 4o	**-**	1.000 (n.s.)	0.899 (n.s.)	1.000 (n.s.)
Google Gemini	-	-	1.000 (n.s.)	0.783 (n.s.)
Claude 3.5 Sonnet	-	-	-	1.000 (n.s.)
Mistral Large 2	-	-	-	-
**MRI -** Contrast media information				
ChatGPT 4o	**-**	0.544 (n.s.)	1.000 (n.s.)	0.544 (n.s.)
Google Gemini	-	-	0.353 (n.s.)	1.000 (n.s.)
Claude 3.5 Sonnet	-	-	-	0.544 (n.s.)
Mistral Large 2	-	-	-	-
**MRI -** Pregnancy, breastfeeding and pediatric examinations				
ChatGPT 4o	-	1.000 (n.s.)	1.000 (n.s.)	1.000 (n.s.)
Google Gemini	-	-	1.000 (n.s.)	1.000 (n.s.)
Claude 3.5 Sonnet	-	-	-	1.000 (n.s.)
Mistral Large 2	-	-	-	-
**MRI -** Pre- and post-examination Information				
ChatGPT 4o	-	1.000 (n.s.)	1.000 (n.s.)	1.000 (n.s.)
Google Gemini	-	-	0.944 (n.s.)	1.000 (n.s.)
Claude 3.5 Sonnet	-	-	-	1.000 (n.s.)
Mistral Large 2	-	-	-	-

*: significant ($p < 0.050$); **: $p < 0.010$; n.s.: $p > 0.050$

Supplementary Table S5 Display of Interrater Agreement for both radiologists (Intraclass Correlation Coefficient (ICC)) per model for CT and MRI

	**ICC**	
**Model**	*CT*	*MRI*
ChatGPT-4o	0.006	0.072
Google Gemini	0.012	0.260
Claude 3.5 Sonnet	0.071	-0.026
Mistral Large 2	0.060	0.000

Possible and frequently asked Patient questions before undergoing Computed tomography (CT) and Magnetic resonance imaging (MRI) - Display of each question asked sorted by different categories.

CT questions

General and technical information

- What is a CT scan?
- Are there differences in the quality of CT machines, so that it is important which clinic I go to for my CT?
- What is the difference between CT and MRI?
- What is the difference between CT and PET-CT?
- What is the difference between CT and X-ray?
- How often can I have a CT scan?
- Can a tumor be imaged with a CT scan?
- Can a CT scan be used to visualize a bone fracture?
- What are alternative radiological examination methods to CT?
- What is the maximum duration of a CT scan?
- Is a CT scan painful?
- Is a CT scan possible for patients with claustrophobia?
- Do I have to pay attention to my breathing during the CT scan?

Contrast media information

General Information and side effects

- What is a contrast agent?
- What are common contrast agents in CT and how do they differ?
- When are contrast media necessary in CT?
- Can you feel anything when contrast media is administered in a CT scan?
- What are the possible side effects of contrast media in CT scans and how often do they occur?
- Is a different contrast agent used for CT examinations of the gastrointestinal tract than for other examination regions?

Allergic reaction

- Is there often an allergic reaction or intolerance to contrast media in CT scans?
- I am prone to allergies (to medication, pollen, animal hair) - is there an increased risk of allergic reactions to contrast media in CT scans?
- The last time I had a CT scan I had a mild allergic reaction with tingling - do I need to take anything before the contrast medium is administered this time?
- I have often had contrast media for CT and have never had an allergic reaction - can this still happen?

Extravasation

- What can happen as a complication when placing the venous access for CT contrast agent administration or during the administration itself via the venous access?
- What is an extravasation?
- What are the possible consequences of extravasation due to contrast agents in CT?

Kidney

- Does the contrast medium in the CT have an effect on kidney function?
- I have normal kidney function - can the CT contrast agent restrict this?
- I am prone to kidney and bladder infections and just had a bladder infection last week - am I now at higher risk of kidney failure due to the contrast agent in the CT?
- My kidney function is impaired with a GFR of about 45 - can I have a CT with contrast?
- I have known renal insufficiency and am still on dialysis - can I still have a CT with contrast medium?

- I have known renal insufficiency and will not receive my dialysis until tomorrow - can I still have a CT scan with contrast medium today?

Thyroid gland

- Can contrast media in a CT scan have side effects on the thyroid gland?
- I have Hashimoto's and take L-thyroxine - can I have a CT scan with contrast medium?
- I have hypothyroidism and don't take L-thyroxine, my TSH is above the reference range - can I get a CT with contrast today?
- I have hypothyroidism and take L-thyroxine, my TSH is below the reference range - can I get a CT with contrast today?
- I have well-controlled hyperthyroidism - can I have a CT scan with contrast medium today?
- I have a latent hyperthyroidism - can I have a CT scan with contrast medium today?
- I have manifest hyperthyroidism - can I have a CT scan with contrast medium today?

Pregnancy, breastfeeding and pediatric examinations

- I am taking contraceptives - can the contrast agent in the CT scan interact with them?
- I am pregnant - can I have a CT scan?
- I am pregnant - can I have a CT scan with contrast medium?
- I am breastfeeding and am to be given a contrast agent for the CT - can this have side effects for me or the baby?
- My child is to have a CT scan - do I need to pay attention to anything in particular?
- What are the special side effects that can occur during a CT scan in children?
- Are there different risks regarding radiation exposure in CT scans for children than for adults?
- My child has already had a CT scan this year, is it okay if he has another one?
- Are there different risks for women and children than for men with regard to radiation exposure in CT scans?

Pre- and post-examination Information

- I am due to have a CT scan focusing on the gastrointestinal tract - is there anything special I need to be aware of before the scan?
- What medication may have to be administered beforehand for CT examinations focusing on the stomach, small intestine or large intestine?

- How old can my last kidney values (creatinine and GFR) and thyroid values (TSH, T3, T4) be before the CT scan?
- Do I have to fast before my CT scan?
- Can I do anything before my CT scan to minimize the risk of possible kidney damage?
- Do I have to pay attention to anything in particular after the CT scan?
- Do I have to drink a lot after the CT scan with contrast medium?
- I am to have a whole-body CT scan with contrast medium because of my tumor follow-up - I have high blood pressure and have already had two heart attacks, is this a problem for the CT scan?
- I have a pacemaker - can I have a CT scan with contrast medium?

MRI questions

General and technical Information

- What is an MRI?
- What can you see well in an MRI?
- Are there differences in the quality of MRI machines, so that it is important which clinic I go to for my MRI?
- Are there open build MRI machines?
- What is the difference between MRI and CT?
- What is the difference between MRI and PET-MRI?
- How often can I have an MRI scan?
- Will I be exposed to harmful radiation during an MRI?
- Do I have to pay attention to my breathing during the MRI examination?
- Is it possible to examine several areas of the body at the same time using MRI?
- How long does an MRI scan usually take?
- Is it possible to listen to music during the MRI examination?
- I am very sensitive to loud noises - can I still have an MRI?
- I am claustrophobic - is this a problem during an MRI scan?
- My blood vessels are to be examined - is an MRI better suited to this than a CT scan?
- Can I cross my arms and/or legs during the MRI scan?
- I have several tattoos - can this be a problem if I am to have an MRI?
- Can an MRI scan be dangerous?

- I already had a CT scan today and am now due to have an MRI, is that a problem?
- I have had tinnitus for a long time - can it be made worse by the MRI?
- I am due to have an MRI scan of my breast - is there anything in particular I need to be aware of?

Information about external material

- Can I carry metal objects with me during the MRI examination?
- I have a pacemaker - can I still have an MRI?
- I have a defibrillator - can I still have an MRI?
- I have a dental implant and am unsure whether it is magnetic - can I still have an MRI?
- I have metal in my body due to an operation - can I still have an MRI?
- I had a hip replacement 10 years ago - can I have an MRI?
- Are there also MRI-compatible metals?
- I have had heart surgery - can I still get an MRI?
- I have had brain surgery - can I still have an MRI?
- I wear permanent make-up - can I still get an MRI?
- I wear a transdermal patch - can I still get an MRI?
- I wear a retainer after braces - can I still get an MRI?
- I have a cochlear implant - can I still have an MRI?
- I wear an Intrauterine Device (IUD) for contraception - can I have an MRI?
- I have metal in my body from a previous operation - if possible, is it better to have a 1.5T or 3T MRI scan?
- I have a ventricular shunt - can I have an MRI?

Contrast media information

- What are common contrast agents used in MRI?
- When are contrast agents necessary in MRI?
- What are the differences between the contrast agents used in MRI and those used in CT?
- What are the most common side effects and risks of an MRI scan without and with contrast medium?
- What are the possible effects and side effects of contrast media in MRI?

- Is an allergic reaction to MRI contrast agents common?
- My liver values are normal - can MRI contrast media still damage my liver?
- My liver values are poor - can MRI contrast media cause further damage to my liver?
- I had a liver transplant - can I have contrast agent in the MRI?
- My kidney values are normal - can MRI contrast media still damage my kidneys?
- My kidney values are poor - can MRI contrast agents further damage my kidneys and worsen their function?
- I had a kidney transplant - can I have contrast media in the MRI?
- I am on dialysis - can I have contrast media in the MRI?
- Can MRI contrast media accumulate in the body and if so, where and what are the consequences?

Pregnancy, breastfeeding and pediatric examinations

- I am pregnant - can I still get an MRI?
- I am pregnant - can I have an MRI with contrast medium?
- I am breastfeeding and am to have a contrast agent for an MRI - can this have side effects for me or the baby?
- My child is to have an MRI - is there anything in particular to watch out for in children before and during the examination compared to adults?
- I am taking contraceptives - can the contrast agent in the MRI scan interact with them?
- Why should my child be given anesthesia during the MRI scan?

Pre- and post-examination Information

- Do I have to stay in hospital after the MRI examination with contrast medium or can I go home on my own?
- I need a sedative before the MRI scan - can I still go straight home after the scan?
- Why do I have to drink pineapple juice before my abdominal MRI scan?
- Do I have to be sober for the MRI of the abdomen?
- Why am I not allowed to have any caffeinated drinks or chocolate 12 hours before my heart MRI?
- Can I get something for my claustrophobia before the MRI?
- I am always given sedatives before my MRI scan - is there anything I need to be aware of?

List of abbreviations within all questions

CT	Computed tomography
MRI	Magnetic resonance imaging
PET	Positron emission tomography
1.5T	1.5 Tesla
GFR	Glomerular filtration rate
TSH	Thyroid-stimulating hormone
T3	Triiodothyronine
T4	Thyroxine
IUD	Intrauterine Device
